# Evaluation of two methods for detecting anti-A and anti-B hemolysins in blood donors: a prospective study in Abidjan

**DOI:** 10.11604/pamj.2025.51.56.48075

**Published:** 2025-06-23

**Authors:** Amah Patricia Victorine Goran-Kouacou, Akou Sara Adélaïde Bognini, Yida Jocelyne Séri, Angbonon Tychique Elysée Attoukoula, Aka Ange Aurel Ghislain Krou, Yannick Armand Kouassi, Séry Romuald Dassé

**Affiliations:** 1 Immunology and Hematology Laboratory, Cocody University Hospital, Abidjan, Côte d’Ivoire,; 2 Immunology-Allergology Department, Medical Sciences Training and Research Unit, Félix Houphouët-Boigny University, Abidjan, Côte d'Ivoire,; 3 Hematology Biology Department, Medical Sciences Training and Research Unit, Félix Houphouët-Boigny University, Abidjan, Côte d'Ivoire

**Keywords:** Hemolysins, anti-A and anti-B antibodies, functional hemolysis test, indirect agglutination, transfusion safety, Côte d’Ivoire

## Abstract

**Introduction:**

anti-A and anti-B hemolysins are IgG antibodies capable of causing intravascular hemolysis during non-ABO-identical transfusions, particularly with plasma-rich components. Their identification in blood donors is crucial for transfusion safety. This study aimed to compare the performance of indirect agglutination and functional hemolysis assays in detecting these antibodies.

**Methods:**

we conducted a prospective comparative study involving 300 voluntary blood donors recruited at Cocody University Hospital between January and March 2025. Each sample was tested using two parallel methods: (1) an indirect agglutination assay performed after thermal inactivation of IgM antibodies; and (2) a functional hemolysis test employing guinea pig complement. Hemolysin titers were considered significant if ≥32 for agglutination or ≥8 for hemolysis. Multivariable logistic regression analysis was used to identify independent factors associated with a positive hemolysis test result.

**Results:**

agglutination detected 54 positive cases (18.0%), and hemolysis identified 30 cases (10.0%). Significant titers were found in 7.4% (agglutination) versus 60.0% (hemolysis) of positive cases. Approximately 70% of positive donors were blood group O with either method. Overall concordance between methods was 92% (κ = 0.67). The agglutination method had 100% sensitivity and 91.1% specificity. A history of pregnancy was the only factor significantly associated with a positive hemolysis test (adjusted odds ratio = 2.77; 95% CI: 1.08-7.14; p = 0.033).

**Conclusion:**

the functional hemolysis test more specifically identifies antibodies with potential transfusion risk. In resource-limited settings, targeted screening based on ABO group, antibody titers, and donor immunological history (especially pregnancy) could improve transfusion safety.

## Introduction

Ensuring transfusion safety relies fundamentally on the identification and prevention of immunological incompatibilities, particularly within the ABO blood group system. In the context of non-identical ABO transfusions involving plasma-rich blood components (such as platelets or fresh frozen plasma) IgG anti-A and/or anti-B antibodies present in the donor´s plasma may activate complement at body temperature and lead to acute intravascular hemolysis in susceptible recipients [1-3]. These antibodies, known as hemolysins, usually appear after antigenic stimulation, such as a transfusion or pregnancy [4-6]. They can be found in donors of all ABO groups but are more commonly detected in individuals with blood group O [4-6]. To identify them, two main methods are used in practice. The first is the indirect agglutination test, which involves neutralizing IgM antibodies by heat to detect underlying IgG activity. The second is the functional hemolysis assay, which evaluates whether these antibodies can trigger red cell lysis in the presence of complement. The agglutination technique is simple, rapid, and widely adopted in routine settings. However, it may not accurately reflect the true hemolytic potential of the antibodies.

In contrast, the functional test offers a more physiologically relevant assessment, but requires meticulous technical execution, strict complement handling, and nuanced interpretation, factors that limit its widespread use in resource-limited laboratories [7-10]. Despite its clinical relevance, comparative data on the performance of these two methods remain scarce in sub-Saharan Africa [11-14]. Moreover, there is no consensus on the titer thresholds defining clinically significant hemolysins, particularly in settings where transfusion safety protocols are constrained by technical and economic limitations [1,8,9,15].

This study aimed to fill that gap by comparing the diagnostic performance of indirect agglutination and functional hemolysis assays in detecting anti-A and anti-B hemolysins among voluntary blood donors at Cocody University Hospital. Beyond measuring concordance between the methods, we sought to identify donor-related factors associated with hemolysin positivity in order to inform a more selective and context-appropriate screening strategy.

## Methods

**Study design and setting:** this prospective and comparative study was conducted from January to March 2025 at Cocody University Hospital in Abidjan, Côte d´Ivoire. The study took place in the hospital´s Immunology and Hematology Laboratory, in collaboration with the blood collection unit. Cocody University Hospital routinely carries out donor recruitment and qualification activities. The affiliated laboratory performs immunohematological and biological analyses for diagnostic purposes, clinical monitoring, and transfusion safety.

**Study population:** voluntary blood donors aged 18-60 years who met national eligibility criteria and provided written informed consent were enrolled at the Cocody University Hospital collection site during the study period. Donors with autoimmune diseases, recent acute infections, or current immunosuppressive therapy were excluded. Donors with blood group AB were also excluded to avoid detection of passive anti-A or anti-B antibodies from previous transfusions.

**Figure 1:**
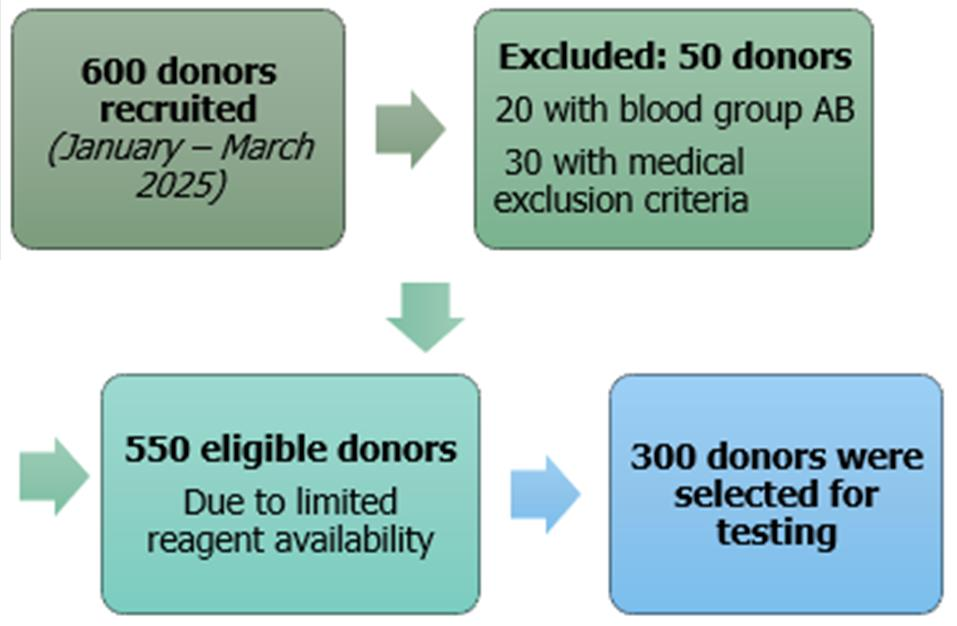
flow diagram of donor selection and inclusion

**Sample size:** a convenience sample of consecutive donors was used. Based on recruitment capacity and available resources, we aimed to include 300 donors (Figure 1).

**Data collection:** a standardized questionnaire was used to collect sociodemographic data (age, sex), immunohematological data (ABO blood group, transfusion history, pregnancy history), and donation characteristics (first-time or repeat donor).

**Laboratory analysis:** two techniques were applied in parallel to detect anti-A and anti-B hemolysins.

**Indirect agglutination method:** in a test tube, donor serum was diluted 1:3 in 0.9% saline and heated at 56°C for 30 minutes to inactivate IgM while preserving IgG antibodies [10,16,17]. Test red blood cells (A1 and B, RhD-negative) were treated with papain to enhance antigenic reactivity. The heated serum was then incubated with the red cells for 45 minutes at 37°C [17]. After centrifugation (1000 rpm for 1 minute), visible agglutination was recorded as a positive result. Titration was performed by serial dilution (1:2, 1: 4, 1:8, etc.). The titer was defined as the highest dilution showing visible agglutination. A titer ≥32 was considered clinically significant [4,8,9].

**Functional hemolysis test:** in a test tube, 25 μL of unheated donor serum was mixed with 25 μL each of papain-treated A1 and B test red cells (RhD-negative), and 25 μL of reconstituted guinea pig complement (Sigma-Aldrich, St. Louis, MO, USA), used in accordance with the manufacturer's instructions. This reagent is widely used in hemolysis testing and is recommended in African transfusion protocols [2,17]. The mixture was incubated for 60 minutes at 37°C. After centrifugation (3000 rpm for 5 minutes), the presence of a red-colored supernatant (in the absence of agglutination) was considered indicative of hemolysis. Titration was performed by serial dilution (1:2, 1:4, 1:8, etc.), and the functional titer was defined as the highest dilution showing visible hemolysis. A titer ≥8 was considered indicative of clinically significant hemolytic activity [1,8].

Both detection methods were applied to each sample. Results were compared based on the presence or absence of hemolysins, antibody titers, and antibody specificity (anti-A, anti-B, or both). In accordance with Association for the Advancement of Blood & Biotherapies (AABB) and Joint United Kingdom (UK) Blood Transfusion and Tissue Transplantation Services Professional Advisory Committee (JPAC) guidelines, the functional hemolysis test was considered the reference standard for assessing transfusion-related hemolytic risk [7,15,18].

**Variables:** we recorded the following variables: sociodemographic (age, sex); immunohematological (ABO blood group, detection method, antibody specificity (anti-A, anti-B, or combined), antibody titer); and clinical history (transfusion history, pregnancy history, type of donation).

**Statistical analysis:** data were analyzed using SPSS (version 26.0). Categorical variables are presented as counts and percentages. Continuous variables are presented as the mean ± standard deviation. Group comparisons were performed using the Chi-squared test or Fisher´s exact test, as appropriate. Agreement between the two methods was assessed using Cohen´s kappa coefficient. Sensitivity, specificity, positive predictive value, and negative predictive value were calculated with 95% confidence intervals. Age was analysed both as a continuous variable and as a binary variable categorized at 35 years (≤35 years vs >35 years) for regression analysis. A p-value < 0.05 was considered statistically significant.

**Ethical considerations:** our study received approval from the Ethics Committee of Cocody University Hospital (ref. no. 122/MSHP-CMU/CHU-C/DMS/RK/25). All participants gave written informed consent before enrollment. Measures were taken at every stage to ensure strict confidentiality and protect donor privacy.

## Results

**Participants and selection:** a total of 600 blood donors were recruited. After excluding 50 donors (20 with blood group AB and 30 for medical reasons), 550 donors remained eligible. Due to limited reagent availability, only 300 donors were tested using both the agglutination and hemolysis methods (Figure 1).

**General characteristics:** the 300 included donors were predominantly male (n = 243; 81.0%) with a mean age of 29.2 ± 8.6 years (range 18-55 years). The ABO blood group distribution was as follows: group O, 174 donors (58.0%); group A, 65 donors (21.7%); group B, 61 donors (20.3%).

**Figure 2:**
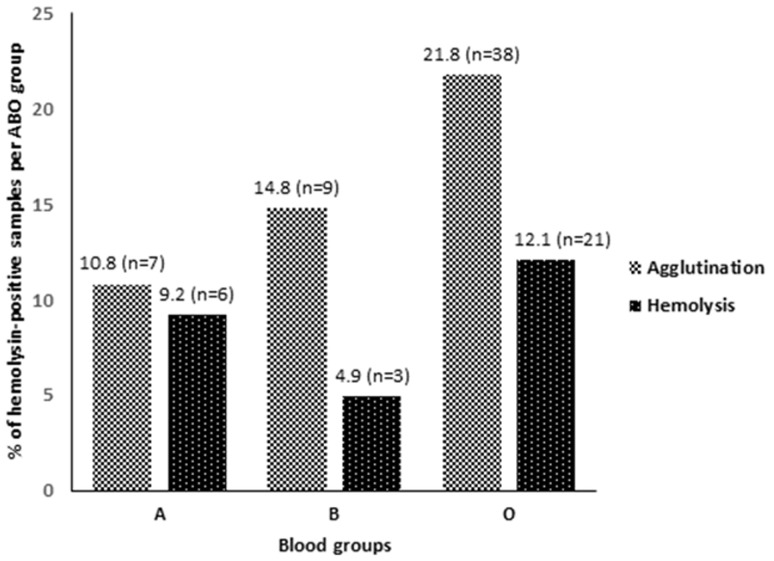
comparison of hemolysin detection rates by ABO group and method

**Hemolysin detection:** the agglutination method detected hemolysins in 54 donors (18.0%; 95% CI: 13.8-22.2), whereas the hemolysis test identified hemolytic activity in 30 donors (10.0%; 95% CI: 6.6-13.4). The difference in detection rates between the two methods varied by ABO group, with differences of +1.6% in group A, +9.9% in group B, and +9.7% in group O (Figure 2).

**Hemolysin specificity:** group O donors accounted for most positive cases (70.4% by agglutination; 70.0% by hemolysis). Antibody specificity profiles were broadly similar between methods (Table 1). Among group O donors, anti-A antibodies were observed in 27.8% (agglutination) versus 20.0% (hemolysis); anti-B antibodies in 16.7% versus 23.3%; and combined anti-A and anti-B in 25.9% versus 26.7%. In group A donors, anti-B antibodies were found in 13.0% (agglutination) and 20.0% (hemolysis); in group B donors, anti-A antibodies were found in 16.7% (agglutination) versus 10.0% (hemolysis) (Table 1). Chi-squared analysis showed significant differences in antibody specificity distribution by ABO group for both agglutination (χ^2^= 30.29; p < 0.0001) and hemolysis (χ^2^= 16.37; p = 0.0026).

**Table 1:** specificity of anti-A and anti-B hemolysins by ABO group and detection method

ABO group	Anti-A n (%)	Anti-B n (%)	Anti-A and anti-B n (%)	Total n (%)
**Agglutination (n=54)**				
A	0 (0.0%)	7 (13.0%)	0 (0.0%)	7 (13.0%)
B	9 (16.7%)	0 (0.0%)	0 (0.0%)	9 (16.7%)
O	15 (27.8%)	9 (16.7%)	14 (25.9%)	38 (70.4%)
**Hemolysis (n = 30)**				
A	0 (0.0%)	6 (20.0%)	0 (0.0%)	6 (20.0%)
B	3 (10.0%)	0 (0.0%)	0 (0.0%)	3 (10.0%)
O	6 (20.0%)	7 (23.3%))	8 (26.7%)	21 (70.0%)

**Distribution of hemolysin titers:** high antibody titers were more frequently detected by the functional test. The functional test identified titers ≥8 in 60.0% of hemolysis-positive cases, compared to titers ≥32 in only 7.4% of agglutination-positive cases (χ^2^= 24.94; p < 0.0001) (Table 2). In the agglutination-positive group, titers ≥32 were observed in three group O donors (13.0%) and one group B donor (5.9%); no group A donor had a significant titer. In the hemolysis-positive group, titers ≥8 were observed in 13 group O donors (61.9%), 3 group A donors (50.0%), and 2 group B donors (66.7%) (Table 2). Fisher´s exact test showed no significant differences in titer distribution by ABO group for either method (agglutination: p = 0.2748-1.000; hemolysis: p = 0.6618-1.000).

**Table 2:** distribution of hemolysin titers by blood group and detection method

Agglutination	A (n=14)	B (n=17)	O (n=23)	Total (n=54)
< 32 (non significant)	14 (100%)	16 (94.1%)	20 (87.0%)	50 (92.6%)
≥ 32 (significant)	0 (0.0%)	1 (5.9%)	3 (13.0%)	4 (7.4%)
**Hemolysis**	**A (n=6)**	**B (n=3)**	**O (n=21)**	**Total (n=30)**
< 8 (non significant)	3 (50.0%)	1 (33.3%)	8 (38.1%)	12 (40.0%)
≥ 8 (significant)	3 (50.0%)	2 (66.7%)	13 (61.9%)	18 (60.0%)

**Concordance between methods:** overall, there was 92.0% agreement between the two techniques, with Cohen´s κ = 0.67 indicating substantial agreement. No false-negative results were observed. The agglutination method demonstrated 100% sensitivity (95% CI: 88.6-100) and 91.1% specificity (95% CI: 87.1-94.0). Its positive predictive value was 55.6% (95% CI: 42.4-68.0) and its negative predictive value was 100% (95% CI: 98.5-100) (Table 3).

**Table 3:** concordance between agglutination and functional hemolysis tests

Agglutination vs Hemolysis	Hemolysis +	Hemolysis -	Total
Agglutination +	30	24	54
Agglutination -	0	246	246
Total	30	270	300

**Factors associated with functional positivity:** in bivariate analysis, no significant association was found between hemolysis positivity and age, sex, ABO group, or transfusion history. Only pregnancy history was significantly associated with a positive hemolysis test (p = 0.038) (Table 4). In multivariable logistic regression, pregnancy remained significantly associated with hemolysis positivity (adjusted OR = 2.77; 95% CI: 1.08-7.14; p = 0.033) (Table 5).

**Table 4:** bivariate analysis of factors associated with positive hemolysis test

Explored factor	p-value
Sex (male vs female)	1.000
ABO group	0.272
History of transfusion	0.393
Previous pregnancy	0.038
Age > 35 years	0.081
First-time vs regular donor	0.150

**Table 5:** multivariable logistic regression of predictors of functional hemolysis

Variable	Regression coefficient	Standard error	z	p-value	OR	IC 95 %
Intercept	-2.15	0.48	-4.48	< 0.001	-	-
Sex	-0.10	0.50	-0.20	0.840	0.90	0.34-2.36
Transfusion history	0.30	0.52	0.58	0.561	1.35	0.49-3.76
Previous pregnancy	1.02	0.48	2.13	0.033	2.77	1.08-7.14
Age > 35 years	0.65	0.42	1.55	0.121	1.91	0.84-4.35
First-time donor	0.28	0.47	0.60	0.552	1.32	0.52-3.35
B group (vs A)	-0.55	0.68	-0.81	0.418	0.58	0.15-2.18
O group (vs A)	0.47	0.51	0.92	0.357	1.60	0.58-4.41

## Discussion

In this study, we compared the performance of indirect agglutination and functional hemolysis assays to detect anti-A and anti-B hemolysins in blood donors, with the goal of identifying transfusion-risk antibodies in an African hospital setting. We detected a higher proportion of positive sera with the agglutination test (18.0%) than with the functional hemolysis test (10.0%). However, among the agglutination-positive samples, only 7.4% reached a clinically significant titer, compared to 60.0% of the samples that tested positive in the hemolysis assay. Group O donors accounted for the most positive cases in both techniques (approximately 70%). The overall agreement between methods was substantial (κ = 0.67; 92.0%). Pregnancy history emerged as the sole variable significantly associated with hemolytic activity in multivariate analysis. From a methodological perspective, IgM antibodies were neutralized by heat treatment at 56°C for 30 minutes combined with a 1:3 dilution, a protocol designed to eliminate naturally occurring IgM that typically induce strong agglutination reactions but are generally non-pathogenic in vivo. This step also mitigates nonspecific reactivity related to increased protein concentration following heating. Such precautions are critical for the specific detection of IgG hemolysins, which are clinically relevant in the setting of ABO-incompatible transfusions [7,16]. To enhance antibody binding, test red blood cells were treated with papain, an enzyme that reduces membrane sialic acid content and thereby increases antigen exposure and test sensitivity [17]. In contrast to the agglutination assay, the functional hemolysis test was performed using fresh, unheated serum in order to maintain the activity of both IgM and IgG antibodies. This setup more closely mimics physiological conditions and provides a direct assessment of the antibody´s ability to trigger complement activation and induce lysis of mismatched red blood cells. Guinea pig complement was chosen for its well-documented efficacy in activating the classical complement pathway, with better consistency and higher lytic potential than human serum [2,17].

To define clinically relevant reactivity, we adopted threshold values of ≥32 for agglutination and ≥8 for hemolysis, based on published data indicating that titers below these levels rarely result in transfusion-related hemolysis [1,8,9]. In designing the study cohort, individuals with blood group AB were deliberately excluded to eliminate potential confounding from passively acquired antibodies due to prior transfusions. Including such donors could have complicated the interpretation of true endogenous hemolysin profiles [7,16]. This approach is consistent with current expert recommendations, which encourage more targeted screening based on individual immunological risk factors [18]. The higher positivity rate observed with the agglutination method likely reflects its broad sensitivity to IgG anti-A and anti-B antibodies, regardless of their true hemolytic potential [4,5]. Its excellent negative predictive value (100%) reinforces its role as a valuable first-line test. However, the moderate positive predictive value (55.6%) suggests that it may overestimate the actual risk of clinically relevant hemolysis. In contrast, the functional hemolysis test more specifically detects antibodies capable of activating complement at 37°C [1,2], reinforcing its role as a confirmatory assay, particularly relevant when transfusing plasma-rich products from ABO non-identical donors. This approach is consistent with current guideline recommendations [7,15] and with reported cases of acute hemolytic reactions following incompatible platelet transfusions, which highlight the need for robust screening protocols [3,19,20].

Mitigation strategies, such as the use of platelet additive solutions (PAS-F), have shown partial efficacy in reducing antibody titers [7]. Nevertheless, the lack of an international standard for the functional hemolysis assay (including harmonized protocols and interpretation thresholds) remains a major barrier to its broader implementation, especially in resource-limited settings [8,9]. The distribution of antibody specificities by ABO group was consistent with immunological expectations: group O donors produced anti-A, anti-B, or both; group A donors produced anti-B only; group B donors produced anti-A only [5,8,11,17,19,21-23]. The similarity in specificity profiles between methods reinforces the biological validity of the results. The low prevalence of high-titer hemolysins by agglutination (≥32) is consistent with previous reports from Côte d´Ivoire [6], Tunisia [19], Cameroon [11], Malaysia [21], and Brazil [8], where strongly reactive IgG hemolysins are uncommon (<5% of donors). We observed no significant differences in titer distribution across ABO groups, suggesting a similar immune response to environmental stimuli in this population. The strong association between pregnancy history and hemolysis positivity (adjusted OR 2.77) highlights the role of feto-maternal alloimmunization in generating clinically relevant antibodies [12,24], indicating that multigravida donors may carry a higher risk.

**Limitations:** our study has a few limitations: first, the functional hemolysis test is qualitative and relies on visual reading, which can vary slightly between operators. Without photometric measurement, mild or partial hemolysis could go unnoticed, potentially reducing the test´s sensitivity. Secondly, since the study was conducted in a single center, the generalizability of the results to other settings may be limited. Thirdly, we did not analyze IgG subclasses (such as IgG1 or IgG3), which could have provided more detailed information on the hemolytic potential of the antibodies detected. Finally, as no post-transfusion clinical follow-up was carried out, we were unable to establish a link between test results and transfusion outcome.

**Relevance and perspectives:** although donor qualification is typically managed by the National Blood Transfusion Centre, our findings suggest a potential role for targeted screening of at-risk donors at the point of collection. In particular, identifying blood group O donors with high antibody titers or multigravida female donors could support a policy of directing these donors´ contributions to red cell production only, avoiding the use of their plasma for platelet or plasma products. This targeted approach could enhance transfusion safety in resource-limited settings. Enhanced coordination between blood collection centers and transfusion services is needed to implement such strategies, and multicenter studies with clinical outcomes are required to validate these recommendations.

## Conclusion

By directly comparing serological and functional assays, this study highlights the importance of integrating both approaches to better evaluate the risk of transfusion-related hemolysis. The observed link between functional hemolysis positivity and pregnancy history underscores the need to consider donors’ immunological history during screening. These findings support a targeted screening policy that incorporates ABO group, antibody titers, and immunological risk factors, balancing transfusion safety with operational feasibility in low-resource settings.

### What is known about this topic

- Anti-A and anti-B hemolysins are IgG antibodies that can cause intravascular hemolysis during non-ABO-identical transfusions;
- Agglutination is commonly used in routine practice to detect these antibodies, although it may overestimate their hemolytic potential;
- The functional hemolysis test is considered the reference method for evaluating actual lytic activity.

### What this study adds

- This study provides a direct comparison of two hemolysin detection methods in a cohort of blood donors in West Africa;
- It demonstrates that the functional test more specifically identifies clinically relevant hemolytic antibodies;
- It also proposes a targeted screening strategy based on ABO group, antibody titers, and immunological history.
